# Human whole blood influences the expression of *Acinetobacter baumannii* genes related to translation and siderophore production

**DOI:** 10.1371/journal.pone.0326330

**Published:** 2025-07-24

**Authors:** Hannaneh Ghavanloughajar, Moamen M. Elmassry, Amanda M. V. Brown, Abdul N. Hamood

**Affiliations:** 1 Department of Biological Sciences, Texas Tech University, Lubbock, Texas, United States of America; 2 Department of Molecular Biology, Princeton University, Princeton, New Jersey, United States of America; 3 Department of Immunology & Molecular Microbiology, Texas Tech University Health Sciences Center, Lubbock, Texas, United States of America; 4 Department of Surgery, Texas Tech University Health Sciences Center, Lubbock, Texas, United States of America; Cornell University, UNITED STATES OF AMERICA

## Abstract

*Acinetobacter baumannii* is a major cause of bloodstream infections, yet its adaptation and survival mechanisms in human blood remain poorly understood. While previous studies focused on individual blood components, the impact of human whole blood on *A. baumannii* gene expression has not been explored. To address this, we used an ex vivo model where *A. baumannii* was grown in human whole blood from healthy volunteers (WBHV) and compared its gene expression to that in Luria-Bertani (LB) broth using RNA-seq. Our lab has previously employed a similar WBHV vs. LB comparison in *Pseudomonas aeruginosa*, validating this approach. Our results showed that ribosome biogenesis was the most upregulated pathway in WBHV, with 51 out of 55 ribosomal protein genes exhibiting increased expression. We then examined virulence related genes and found upregulation in iron and zinc acquisition systems (*acinetobactin, znuABC*) and biofilm/quorum sensing regulators, including the *csu* operon. Given these findings, we hypothesized that WBHV exposure enhances virulence. Using the *Galleria mellonella* infection model, we confirmed that *A. baumannii* caused higher larval mortality when grown in WBHV than when grown in LB. Upregulation of the *csu* operon, involved in pili assembly, led us to investigate twitching motility, where we observed a significant increase in WBHV. Additionally, since *A. baumannii* exhibits high drug resistance through the regulation of various outer membrane proteins (OMPs), we analyzed OMP expression in response to WBHV. SDS-PAGE and LC-MS/MS analysis identified three OMPs—Omp33–36, CarO, and OmpA—that were downregulated in WBHV. As these proteins mediate carbapenem uptake, we tested imipenem resistance using a minimum bactericidal concentration (MBC) assay and found that WBHV exposure increased *A. baumannii*’s MBC to imipenem, suggesting reduced susceptibility. Our findings provide valuable insights into the adaptive mechanisms of *A. baumannii* in human whole blood, highlighting potential targets for combating its persistence and antibiotic resistance in bloodstream infections.

## Introduction

*A. baumannii*, a multidrug-resistant (MDR) nosocomial pathogen, is known to cause various severe infections such as pneumonia, bacteremia, meningitis, and wound infections. Reports of carbapenem-resistant *A. baumannii* (CRAB) strains have become increasingly common [1,2]. In addition to its resistance capabilities, *A. baumannii* exhibits inherent adaptability to varying environments. For example, when exposed to different human fluids or proteins, it can alter the expression of numerous genes essential for its persistence and survival [3–6]

However, the impact of human whole blood on *A. baumannii*’s gene expression and virulence factors remains unclear. Human whole blood is a rich reservoir of essential nutrients that pathogens depend on. To prevent infection by pathogenic organisms, humans and other mammals employ a process known as nutritional immunity [7]. Initially, this term described the host’s ability to limit the availability of iron (Fe). However, nutritional immunity can also include mechanisms that restrict access to other vital transition metals or use their toxicity to combat microbial invaders. Nutritional immunity of human whole blood generally creates a stress-inducing environment for growing bacteria [7].

While studies using animal models, such as the murine infection model [8], have provided significant insights into *A. baumannii*’s virulence factors and pathogenesis in response to murine whole blood, there are fundamental differences in immune function between species, and insights gained from mouse studies often cannot be directly applied to humans [9]. One of the main differences between human and mouse immune systems, is in innate immunity which is necessary for survival during the initial days after infection until the adaptive immune responds. Because mice and humans encounter distinct groups of pathogens, their innate immune systems have evolved differently [10]. This is significant because the innate immune system triggers rapid and adaptive responses, making it challenging to model human immune reactivity to vaccines, pathogens, and diseases in mice [11]. Ideally, the most relevant data would stem from analyzing bacterial gene expression throughout the course of infections in patients. However, conducting such studies would be exceedingly challenging.

To circumvent this limitation, and as a first step to better understand the host-bacterium interaction of *A. baumannii* in human whole blood, we performed the current study, focusing on RNA-seq and complementary experiments to assess changes in this pathogen between lysogeny broth (Luria-Bertani) (LB) and human whole blood from healthy volunteers (WBHV). Our study design follows a previously published study from our lab, in which *P. aeruginosa* strain PAO1 was grown in human whole blood from healthy volunteers and LB, and its transcriptomic response was analyzed [12]. By adopting a similar approach, we aim to discover important and critical physiological changes in *A. baumannii* in response to human whole blood, providing insights into its role in nosocomial infections.

Focusing on *A. baumannii* strain A118, isolated from a blood culture of an ICU patient [13], we investigated its global gene expression in WBHV. Our study revealed key transcriptomic changes, including alterations in ribosome expression, iron and zinc acquisition, and biofilm-related genes. To explore the impact of WBHV at the phenotypic level, we conducted complementary experiments, including *G. mellonella* infection assays, OMPs analysis, imipenem minimal bactericidal concentration assessments, and twitching motility evaluations.

## Materials and methods

### Ethics statement for collection of WBHV

This study (RB number L21-156) was approved by the Texas Tech University Health Sciences Center Lubbock Institutional Review Board on 12/07/2021 and remained open until all study activities, including data analysis, were completed. The study was closed to accrual on 22/10/2024. Blood samples from healthy volunteers were collected in two recruitment periods: the first from 12/07/2021–31/08/2021 and the second from 05/06/2024–24/09/2024.

Informed written consent was obtained from healthy volunteers (HVs) at University Medical Center, Lubbock, TX, in compliance with ethical practices. HVs were aged 18–89, with no acute or chronic medical conditions. Blood samples were collected by CRI staff through venipuncture as per the IRB-approved protocol. Blood was collected within 72 hours of admission. A total of 25 mL of blood was collected from each person into three BD Vacutainer tubes (Becton Dickinson, Franklin Lakes, NJ, USA), containing

0.35% sodium polyanethole sulfonate as an anticoagulant in 1.7 mL of 0.85% sodium chloride (SPS). Per the IRB protocol, blood samples were de-identified and were given unique numbers by the CRI staff before the samples were sent to the research laboratory. All methods performed on the samples were in compliance with the relevant guidelines and regulations of the IRB-approved protocol.

### Bacterial strain, culture conditions and growth curves

*A. baumannii* A118 is a bloodstream isolate obtained from a patient in an intensive care unit [13–15] and it was purchased from ATCC culture collection (GenBank: CP059039.1, ATCC: BAA-2093 ™). Four *A. baumannii* A118 mutant strains (Δ*pilA*, Δ*pilQ*, Δ*pilT* and Δ*comM*) were generously provided by Dr. Blokesch (Blokesch Lab, Laboratory of Molecular Microbiology, Global Health Institute, School of Life Sciences, Ecole Polytechnique Fédérale de Lausanne (EPFL)) [16]. Bacteria were routinely grown overnight and maintained at 37°C in LB, prior to subculturing bacterial pellets into WBHV and LB in 1:100 ratio. Bacterial growth of 3 independent replicates was monitored by measuring colony-forming units (CFU) every 2 hours over an 8-hour period.

### Growth of *A. baumannii* A118 in WBHV and subsequent bacterial isolation

Whole blood samples were collected from three volunteers. For each volunteer, three BD Vacutainer tubes were collected and pooled into one tube immediately after collection to minimize red blood cell (RBC) lysis. From each volunteer’s blood, three separate 7.5 mL aliquots were used to inoculate three separate 25 mL flasks as technical replicates. Each flask was inoculated with 10⁶ CFU/mL of an overnight LB culture of *A. baumannii* A118 and incubated in a 37°C shaking water bath. At 4 hours post-inoculation (HPI), when bacterial growth reached ~10⁸ CFU/mL, samples were diluted 1:1 with PBS and layered over a Ficoll-based lymphocyte separation medium (Lonza, Basel, Switzerland). The layers containing lymphocytes and granulocytes were discarded, while the RBC and bacterial pellet was treated repeatedly with erythrocyte lysis buffer (Qiagen, Hilden, Germany) until only bacterial cells remained. The final bacterial pellet was resuspended in LB broth, treated with RNAprotect (Qiagen, Hilden, Germany), and stored at −80°C for subsequent analysis.

### RNA isolation and sequencing

Bacterial RNA was extracted following cultivation in both media. Bacterial RNA was extracted using the RNeasy Mini Kit (Qiagen, Hilden, Germany) according to the manufacturer’s recommendations followed by an additional on-column DNase treatment to eliminate any remaining traces of genomic DNA. The purified RNA was quantified using a NanoDrop spectrophotometer (Nanodrop Technologies, Wilmington, DE, USA) and the integrity of the RNA was assessed using a TapeStation 2200 (Agilent, Santa Clara, California, USA) following the manufacturer’s instructions. rRNA was depleted from total RNA with Ribo-Zero rRNA removal kit for bacteria (Epicentre Biotechnologies, Madison, WI, USA). Enriched mRNA samples were run on the TapeStation 2200 to confirm reduction of 16S and 23S rRNA. Paired-end RNA-seq libraries were constructed from the rRNA-depleted mRNA using the TruSeq mRNA library preparation kit following the manufacturer’s protocol (Illumina, San Diego, CA, USA). Then, the multiplexed cDNA libraries were sequenced using a HiSeq 2000 Sequencer (Illumina, San Diego, CA, USA).

### RNA-seq data and pathway analysis

Paired-end reads of each sample were mapped to the *A. baumannii* A118 reference transcriptome (NCBI RefSeq assembly: ASM1467273v1). Mapping was done using the pseudoalignment-based tool, Kallisto [17] with 100 bootstraps per sample for quantification of transcript sequences. Then, Kallisto output files were imported to R software (version 4.2.1) [18] and Pvclust (version 2.2−0) [19] was used for hierarchical clustering of samples, using bootstrap-based pvalues with 10000 bootstrap sample size. DESeq2 (version 1.36.0) [20] was used to normalize transcript counts and test for differential gene expression by the Wald test after integrating the dispersion estimate and fold change estimate from an empirical Bayes approach. The p-values were corrected for multiple testing using the Benjamini-Hochberg procedure [21]. To provide an overview of the entire expression analysis, the expression data of all 14,203 genes with mapped RNA-seq reads were visualized in a plot of log-intensity ratios (M-values) versus log-intensity averages (A-values) (MA plot) (S1 Fig). The MA plot shows both absolute expression intensity of each gene and differences in expression of each gene between groups. Differentially expressed genes (DEGs) were identified based on their corresponding adjusted p-value (padj) ≤ 0.05 and |log2foldchange| ≥ 1.

KEGG pathway analysis was performed by STRING (https://string-db.org/) (accessed on 17/10/2024) [22]. Strength value for each pathway was calculated as Log10(observed/ expected) [23,24].

### Isolation of *A. baumannii* A118 OMPs, SDS-PAGE and LC-MS/MS protein identification

We extracted OMPs following a procedure as previously described [12] with some minor modifications. *A. baumannii* A118 was inoculated into LB and WBHV and incubated for 8 hours. Cultures were centrifuged, washed, and lysed by sonication (10-minute pulses at 50% power, repeated 3 times) using a Kinematica Polytron homogenizer (Kinematica AG, Luzern, Switzerland). The resulting pellet, containing both inner and outer membranes, was resuspended in sterile water, and 1% sarcosyl (Sigma Aldrich, St. Louis, MO, USA) was added to isolate outer membranes by incubating at room temperature with continuous rotation for 1 hour. After ultracentrifugation (Beckman Coulter, Optima XE-90, Brea, CA, USA) at 100,000 × g for 1 hour, the pellet, enriched in OMPs, was resuspended in sterile water. Protein concentration was determined using the Bradford assay (Bio-Rad, Hercules, CA, USA) [25]. Equal amounts of OMPs (30 µg) were separated by SDS-PAGE and stained with Coomassie blue (Sigma-Aldrich, Burlington, MA, USA). OMP bands at 36, 32, and 26 kDa, which were notably repressed in WBHV, were excised, eluted, and identified by LC-MS/MS (Texas Tech University Center for Biotechnology and Genomics).

### Twitching motility

To assess *A. baumannii* A118 and four *A. baumannii* A118 mutant strains’ (Δ*pilA*, Δ*pilQ*, Δ*pilT* and Δ*comM*) twitching motility, Tryptic Soy Agar (TSA) plates with and without 5% sheep blood (Thermo Fisher Scientific, Waltham, MA, USA) were used. Three *pil* gene mutants (*A. baumannii* A118 Δ*pilQ*, Δ*pilA*, and Δ*pilT*), which are lacking genes crucial for twitching motility, were used to evaluate how blood influences twitching motility in relation to these key genes. *A. baumannii* A118 Δ*comM* was used as a control, since the *comM* gene is primarily involved in DNA uptake rather than twitching motility. Each twitching motility plate was stab-inoculated at three distinct sites at the agarose/Petri dish interface using a single isolated bacterial colony per site. Plates were then incubated at 37°C in a humidified chamber for 24 hours. To visualize the bacteria at the interface, agarose was removed from each plate, The plates were rinsed with 1X PBS and stained with 0.1% crystal violet (w/v) (Sigma-Aldrich, Burlington, MA, USA) in water for 5 minutes. To eliminate excess crystal violet, each plate was gently washed with PBS and left to dry. Twitching motility assays were conducted on at least three separate replicates and the motility patterns were quantified by measuring the longest axis of each replicate. Statistical significance (P < 0.05) was determined by ANOVA followed by Tukey’s multiple-comparison test using GraphPad Prism (version 9.5.1) (GraphPad software, San Diego, CA, USA).

### *G. mellonella* infection model

To assess how WBHV-induced changes affect virulence, we used the *G. mellonella* infection model [26]. The *G. mellonella* infection assay was performed as previously described [27] with some modifications. Larvae weighing 200–400 mg that were kept on wood chips in the dark at 4 °C were used for this assay. *A. baumannii* A118 was cultured in either LB or WBHV for 8 hours. The bacterial cultures were diluted in 1X PBS and adjusted to a cell density of 1 × 10⁵ CFU/ml, determined by measuring the optical density at 600 nm. Bacterial inocula were confirmed by plating serial dilutions on LB agar and counting the colony-forming units (CFU) after overnight incubation at 37°C. The experiments were repeated six times with 10 larvae per experimental group, resulting in a total of 60 *G. mellonella* larvae used per condition. Larvae in the control group were injected with 10 µl of sterile 1X PBS, while those in the experimental groups received 10 µl standardized bacterial suspensions prepared from either LB or WBHV cultures. Injections were performed at the last left proleg, and larvae were incubated at 37 °C in sterile Petri dishes. Viability was monitored every 24 hours for a total of 120 hours. Larvae were assessed by their color and gently prodding with a glass rod and those showing no response were considered dead. Survival curves were generated using the Kaplan-Meier method using GraphPad Prism (version 9.5.1) (GraphPad software, San Diego, CA, USA).

### Determination the MBC of imipenem

To determine the MBC of imipenem (MilliporeSigma, Darmstadt, Germany) against *A.* *baumannii* A118, the bacteria were grown overnight, resuspended in 1X PBS, and adjusted to a cell density of 5 × 10⁴ CFU/ml based on the optical density at 600 nm. An equal volume (50 µl) of the bacterial suspension was added to each well of a 24-well microtiter plate containing 1 ml of either LB or WBHV. Antibiotic was added to each well at varying concentrations in triplicates, except in the control replicates, followed by overnight incubation at 37°C in a shaking incubator. The next day, serial dilutions from each well were plated on LB agar, and CFU were counted the following day after incubation at 37°C. The MBC endpoint was defined as the lowest drug concentration that resulted in no visible growth (>99.9% bacterial killing) compared to the corresponding control replicates, after overnight incubation at 37°C, as previously described [28]. The experiments were performed in triplicate.

## Results

### WBHV can sustain the growth of *A. baumannii* A118 *ex vivo*

**Fig 1:**
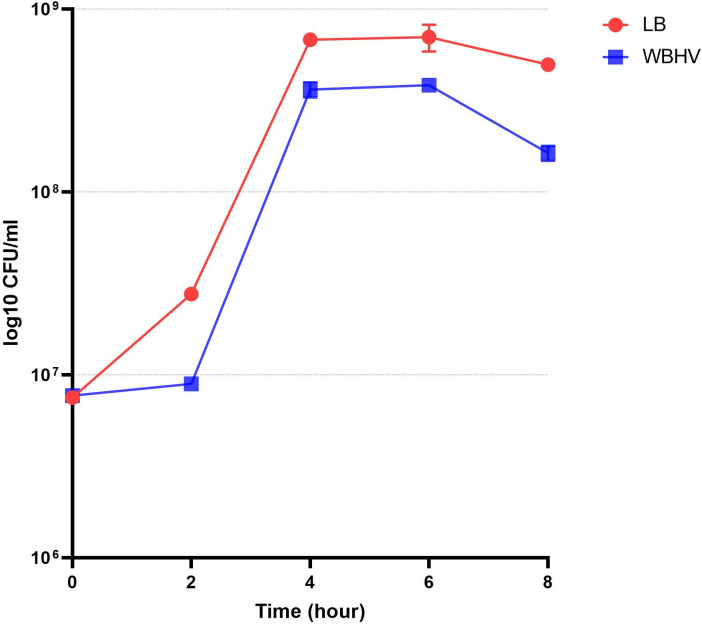
Growth curves of *A. baumannii* A118 grown in either LB or WBHV. Experiments were performed in triplicate.

We first tested how WBHV affected *A. baumannii* A118 growth over time. Results over 8 hours showed *A. baumannii* A118 grew slightly better in LB than in WBHV, reaching higher concentrations at the end of the lag phase and sustaining higher CFU counts throughout the rest of growth period (Fig 1). Both cultures reach optimal growth at 4 HPI, marking the end of the exponential phase. Similarly, both cultures entered the stationary phase at 4 HPI and remained in it until 6 HPI. WBHV cultures exhibited a sharper decline in CFU counts between the 6 and 8 HPI compared to LB cultures.

### Growth of *A. baumannii* A118 in WBHV altered gene expression compared to growth in LB

To test how WBHV affected *A. baumannii* A118 gene expression, RNA was extracted at 4 HPI from both conditions. Based on bacterial growth curves, this time point represented optimal bacterial growth during the exponential phase in both WBHV and LB. Exponential phase is commonly used and recommended for transcriptomic studies, as cells are actively growing and transcribing genes, providing a snapshot of their transcriptional response to the environment.

**Fig 2:**
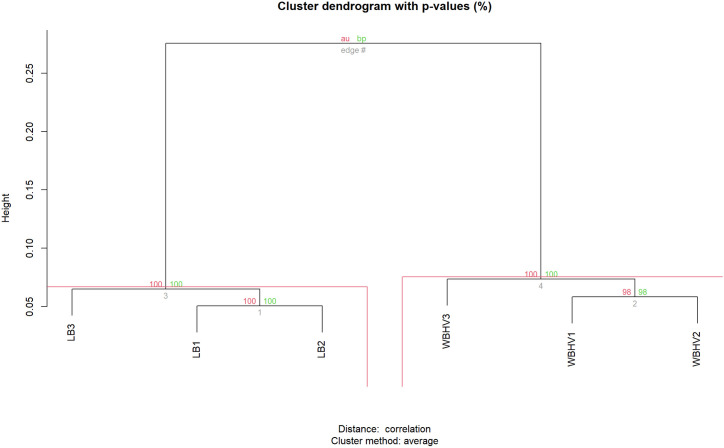
Hierarchical clustering of all biological samples. Values on the edges of the clustering are p-values (%). Red values represent AU p-values, while green values represent BP values. Clusters with AU values greater than 95% are highlighted by rectangles, indicating strong support from the data.

Principal component analysis (PCA) confirmed the close clustering of technical replicates from each volunteer, and clear clustering of biological replicates from each condition after collapsing the technical replicates (S2 Fig). To measure the hierarchical clustering of samples we used two tests, the Approximately Unbiased p-value (AU) and Bootstrap Probability value (BP) (Fig 2). Both tests supported the finding that *A. baumannii* A118’s transcriptome profile was significantly different between LB and WBHV.

**Fig 3:**
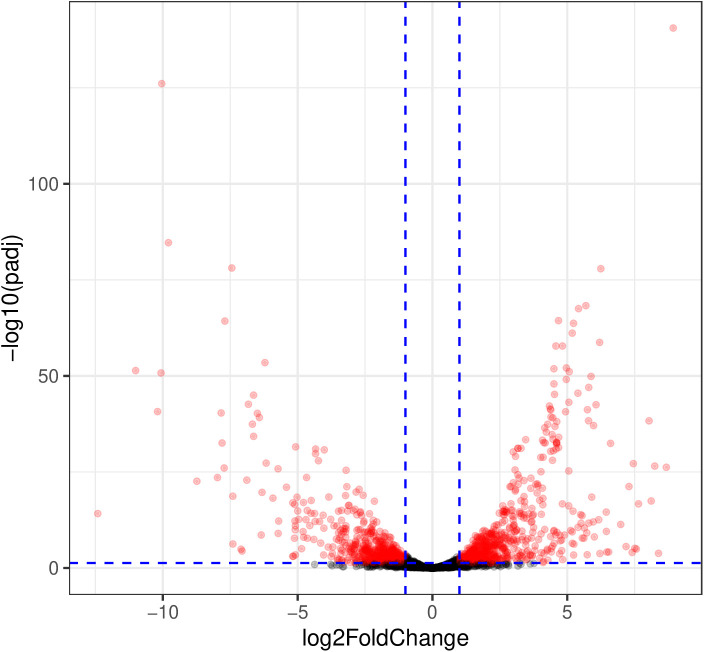
Volcano plots displaying the results of RNA-seq analysis. The dashed blue horizontal line indicates the value of -log10(0.05). Any dot above the dashed blue horizontal line has an adjusted p-value lower than 0.05. The right and left vertical dashed blue lines intercept at x = 1 and x = −1, respectively. Dots to the right of the right line have log2 fold change values greater than 1, while dots to the left of the left line have log2 fold change values less than −1. Genes that are significantly upregulated (adjusted p-value < 0.05, log2FoldChange > 1) are shown with red dots in the upper right quarter of the volcano plot. Genes that are significantly downregulated (p adjusted value < 0.05, log2FoldChange < −1) are shown with red dots in the upper left quarter of the volcano plot.

We found 1297 genes were differentially expressed (padj ≤ 0.05 and |log2foldchange| ≥ 1) between the conditions. Out of these genes, 700 were significantly upregulated in WBHV, whereas 597 genes were significantly downregulated in WBHV

(Fig 3 and S1 Table).

**Fig 4:**
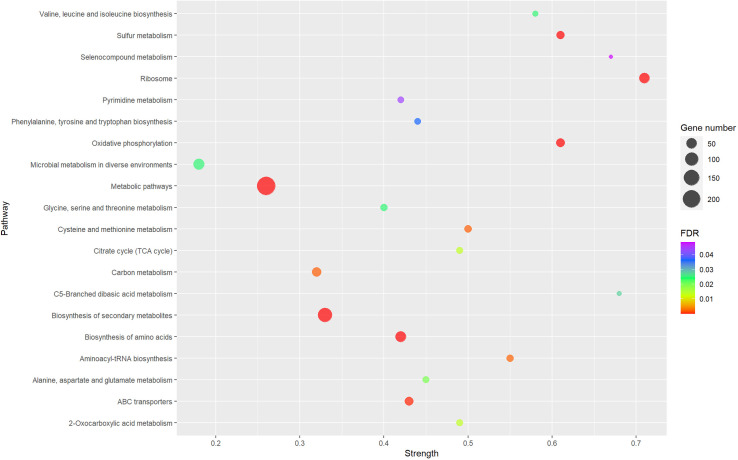
The significantly enriched KEGG pathways within upregulated genes of *A. baumannii* A118 grown WBHV versus LB. Each circle corresponds to a pathway. The circle scale represents the number of differentially expressed genes. The color of the circles represents the FDR value. Strength is calculated by Log10(observed genes/ expected genes). Observed genes are the number of genes in the network that are annotated with a term and expected genes are the number of genes that are expect to be annotated with this term in a random network of the same size.

To investigate the pathways affected by the growth of *A. baumannii* A118 in WBHV, we used STRING KEGG pathway analysis for upregulated genes from RNA-seq analysis, which showed 20 pathways that were significantly enriched among upregulated genes using False Discovery Rate (FDR) ≤ 0.05 (Fig 4). Among the most significantly enriched pathways were ribosome, C5-Branched dibasic acid metabolism, selenocompound metabolism, oxidative phosphorylation, and sulfur metabolism. Ribosome was one of the most significantly enriched pathways in our experiment, with strength value of 0.71 (Fig 4). According to STRING KEGG pathway analysis 51 out of 55 ribosomal protein genes were upregulated in response to WBHV (S3 Fig).

### Many genes involved in iron and zinc acquisition by *A. baumannii*

#### A118 were upregulated by growth in WBHV.

**Fig 5:**
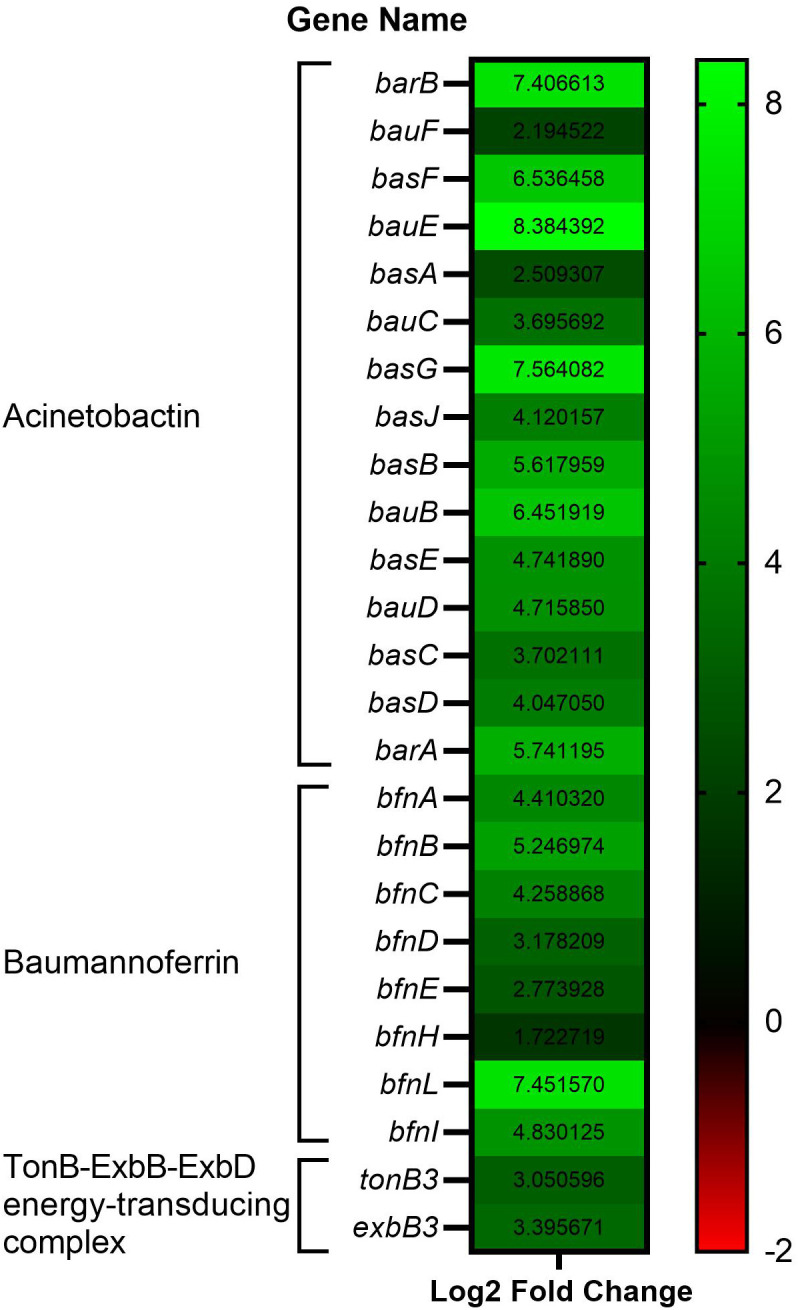
Upregulated genes of *A. baumannii* A118 related to iron acquisition system. In WBHV samples, 15 out of 18 genes involved in acinetobactin biosynthesis and transport, and 7 out of 13 genes related to baumannoferrin biosynthesis and transport, were upregulated. Additionally, *tonB3* and *exbB3* were also upregulated.

Focusing on virulence-related genes, we found that several genes related to iron and zinc acquisition were upregulated. Out of 18 genes involved in acinetobactin biosynthesis, export and import, 15 genes showed upregulation in WBHV samples (Fig 5). Out of 13 genes involved in baumannoferrin biosynthesis, export and import, 8 genes were upregulated in WBHV samples (Fig 5). We identified homologs for all three *tonB* and associated *exbB* and *exbD* genes from the TonBExbB-ExbD energy-transducing complex in *A. baumannii* A118. The genes *tonB3* and *exbB3* were upregulated in WBHV samples (Fig 5). None of the *feo* genes were among the DEGs in our study. Our results showed upregulation of 3 genes from the zinc uptake system (*znuA*, *B* and *D*) involved in zinc transport (S4 Fig).

### Growth of *A. baumannii* A118 in WBHV altered the expression of biofilm and quorum sensing associated genes

One of our questions in this study was how WBHV influences the expression of biofilm and quorum sensing related genes in *A. baumannii* A118. Genes such as *bfmS/bfmR* (two component system), *csuA/BABCDE* (chaperone-usher pili assembly system)*, ata* (*Acinetobacter* trimeric autotransporter)*, ompA* (outer membrane protein A), *pgaABCD* (genes responsible for the production of poly-β-1,6-N-acetylglucosamine), *pglL* (responsible for the glycosylation of multiple proteins), *bap* (biofilm-associated protein) were analyzed separately. Out of these genes, all genes present in the *csu* operon were upregulated in WBHV samples. From the *pga* gene cluster, *pgaC* and *pgaD* were upregulated in WBHV samples. The genes *ata* and *bap* were downregulated in WBHV samples (S5 Fig).

The expression of quorum sensing and quorum quenching related genes such as *acdA* (acyl-CoA dehydrogenase), *fadD* (acyl-CoA synthase), *abaI* (autoinducer synthase), *abaR* (autoinducer receptor), *abaM* (codes for a member of the RsaM protein family)*, ytnP* (lactonase), *aidA* (α/β hydrolase) and two genes encoding lactonase (H0N27_RS07895 and H0N27_RS15615) were analyzed as well. Out of these 9 genes related to quorum sensing and quorum quenching (*acdA, fadD, abaI, abaR, abaM, ytnP, aidA,* H0N27_RS07895 and H0N27_RS15615), we detected transcriptional changes from *abaM* and *ytnP.* In WBHV samples, *abaM* was downregulated, while *ytnP* was upregulated (S5 Fig).

### WBHV affected the OMP profile of *A. baumannii* A118

**Fig 6:**
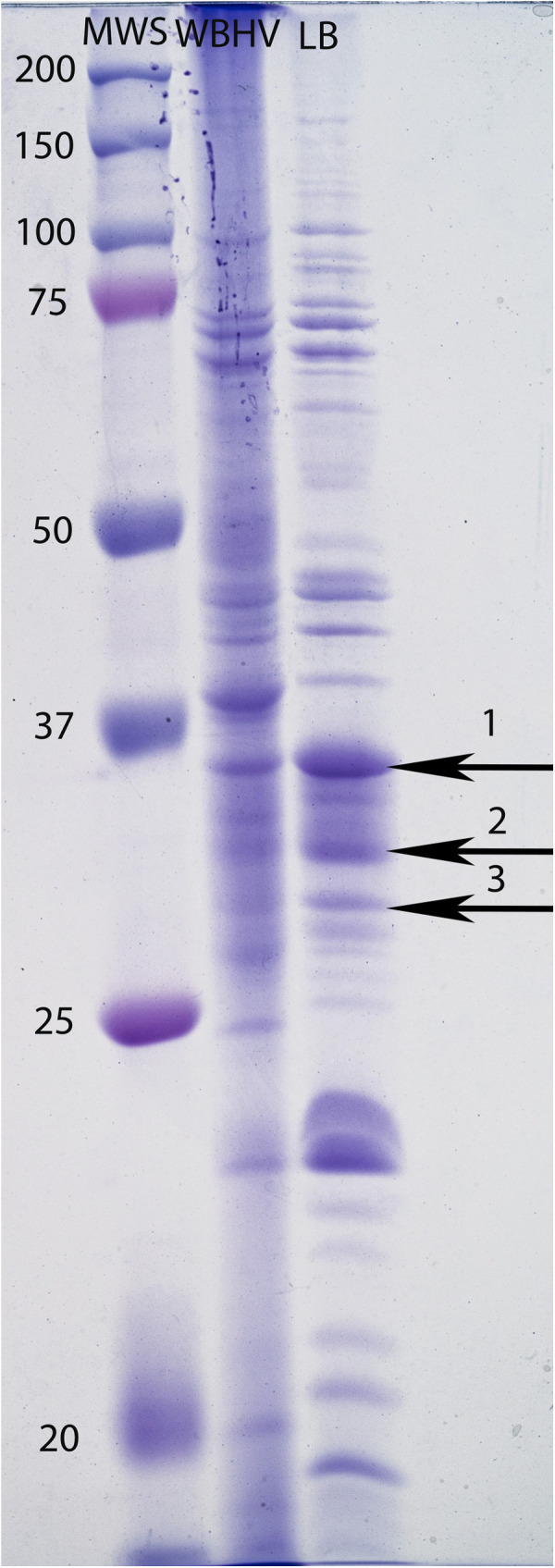
OMP profile of *A. baumannii* A118 grown in WBHV versus LB. Lanes from left to right: MWS (molecular weight standards in KD), OMPs of *A. baumannii* A118 grown in WBHV, OMPs of *A. baumannii* A118 grown in LB.

Given *A. baumannii*’s well-documented multidrug resistance, we sought to determine whether WBHV affects the expression of OMP-related genes involved in antibiotic resistance. OMP extraction was conducted at 8 HPI, representing the late stationary phase. Compared to growth in LB, growth in WBHV induced the production of certain OMPs and reduced the production of others analyzed by SDS-PAGE (Fig 6). We analyzed differentially expressed bands at 25 and 37 kDa range, as several major OMPs in *A. baumannii* have been documented within this range. We detected three bands within this range that showed decreased expression in WBHV but were distinctly upregulated in LB. Mass spectrometry analysis showed that band 1 was Omp38 (24 peptide match, 58% coverage). Band 2 was identified as porin Omp33–36 (3 peptide match, 29% coverage) and band 3 was identified as ornithine uptake porin CarO type 3 (10 peptide match, 48% coverage).

### Sheep blood affected the twitching motility of *A. baumannii* A118

**Fig 7:**
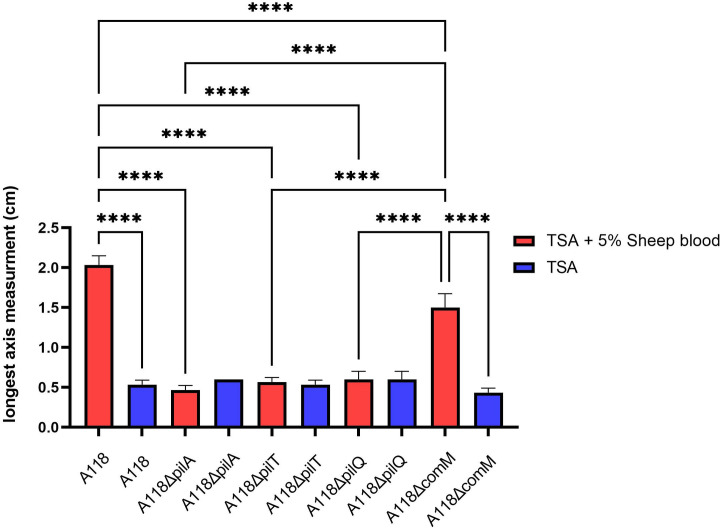
Twitching motility measurements of *A. baumannii* A118, Δ*pilA*, Δ*pilT*, Δ*pilQ* and Δ*comM* on TSA plates with and without 5% sheep blood. Twitching motility of each plate was assessed by measuring the longest axis of the pattern. Twitching motility assays were conducted on at least three separate replicates. Red bars indicate results from TSA twitching plates with 5% sheep blood, while blue bars represent the control TSA plates. Statistical significance (P < 0.05) was determined by ANOVA followed by Tukey’s multiple-comparison test. *, P < 0.05; **, P < 0.01; ***, P < 0.001; ****, P < 0.0001.

Based on our observation of changes in biofilm-related gene expression, specifically upregulation of all *csu* operon genes, we predicted that *A. baumannii* A118’s twitching motility may be affected in response to whole blood. The twitching motility of *A. baumannii* A118 and Δ*comM* on TSA plates containing 5% sheep blood was significantly greater compared to TSA plates without sheep blood. Twitching motility of *A. baumannii* A118 Δ*pilQ*, Δ*pilA*, and Δ*pilT* was not affected significantly by the presence of blood. Additionally, among all strains on TSA plates containing 5% sheep blood, A118 and Δ*comM* twitching motility was significantly greater compared to other tested strains (*A. baumannii* A118 Δ*pilQ*, Δ*pilA* and Δ*pilT*) (Fig 7 and S6 Fig).

### Growth in WBHV increased the killing rate of *A. baumannii* A118 in *G. mellonella*

**Fig 8:**
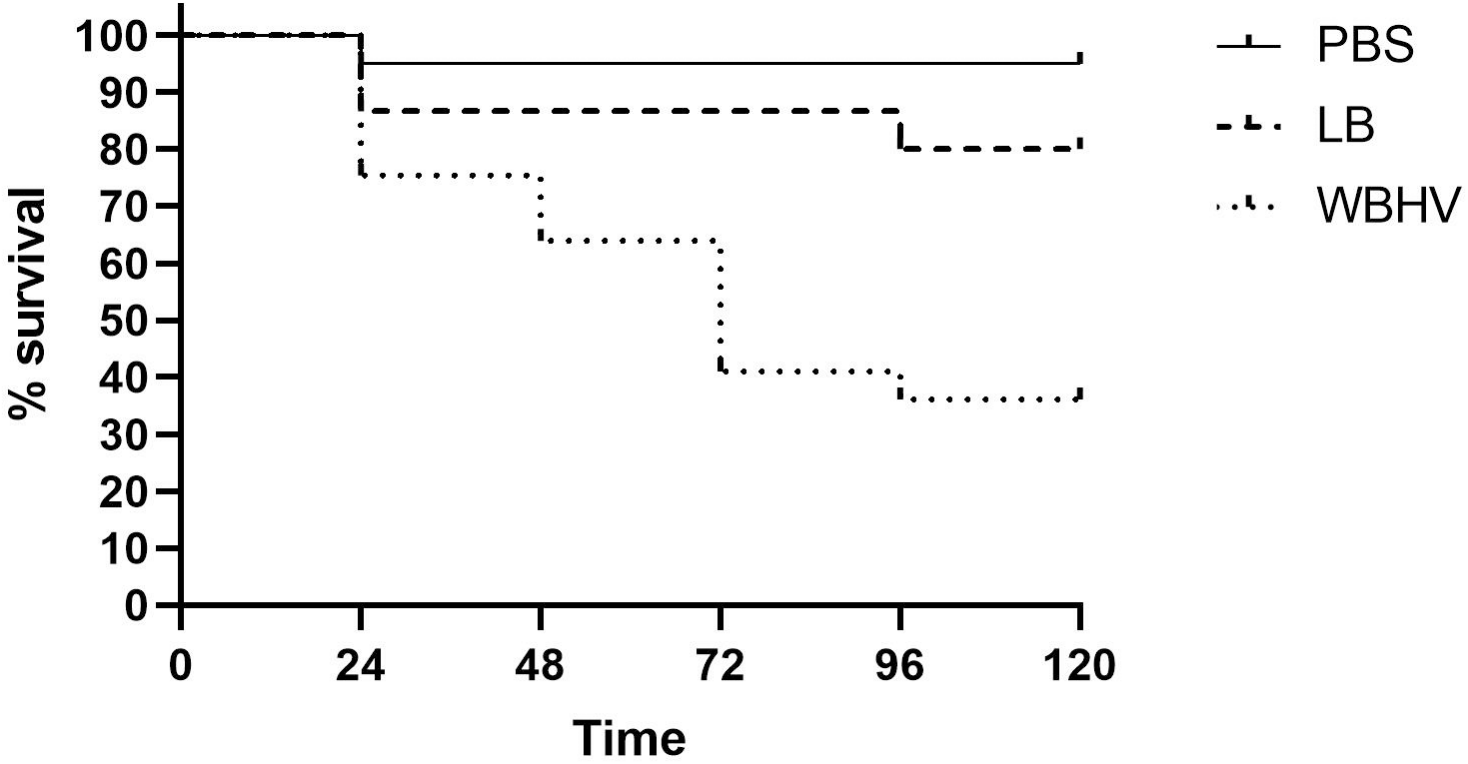
*G. mellonella* survival following infection with *A. baumannii* A118 grown in WBHV or LB. *G. mellonella* infection assays showing differences in survival of larvae injected with 10 µl of a standardized suspension of *A. baumannii* A118 at a concentration of 1 × 10⁵ CFU/ml, after growth in either WBHV or LB, compared to controls injected with sterile 1X phosphate buffered saline (PBS).

Motivated by observation of upregulation of multiple virulence factors in WBHV, we wanted to test whether human whole blood can result in higher mortality rate using the *G. mellonella* infection model. We observed higher mortality rates in *G. mellonella* larvae injected with *A. baumannii* A118 grown in WBHV (Fig 8). After 24 hours, the survival rate of larvae injected with *A. baumannii* A118 grown in WBHV dropped to 75%, while the group injected with an equal number of bacteria grown in LB had a survival rate of 86.6%. Over the next three days, the survival rate of the LB group remained stable, whereas the WBHV group saw a further decline, reaching 36% by day 4. Both groups maintained their respective survival rates through day 5, when the experiment was concluded.

### MBC of imipenem increased when A*. baumannii* A118 was grown in WBHV compared to LB

Based on our observations of changes in gene expression, virulence, and OMP profiles, we examined the changes in *A. baumannii* A118 antibiotic resistance in WBHV compared to LB.

Specifically, the identification of three specific downregulated OMPs (Omp33–36, CarO, and OmpA) in WBHV—all of which play a role in carbapenem resistance, a class of broad-spectrum beta-lactam antibiotics—prompted us to investigate the effects of WBHV on the MBC of the commonly used carbapenem class antibiotic for treating severe infections, imipenem. When *A. baumannii* A118 was grown in LB, a concentration of 10 µg/ml of imipenem was identified as the MBC, achieving >99.9% bacterial killing compared to LB control samples without antibiotic. However, in WBHV, the MBC increased to 50 µg/ml, demonstrating >99.9% bacterial killing compared to WBHV controls. Results were consistent across all three replicates in both LB and WBHV conditions.

## Discussion

*A. baumannii* A118’s virulence mechanisms and responses to human whole blood remain unknown, prompting our investigation. Understanding these responses is critical because of the significant global health threat posed by antimicrobial-resistant ESKAPE pathogens, including *A. baumannii* [2]. The spread of resistance genes among ESKAPE pathogens has led to limited treatment options for severe infections, heightened the disease burden, and raised mortality rates due to treatment failures [29]. We hypothesized that *A. baumannii* A118 would respond to WBHV exposure by upregulating key pathways related to survival, such as virulence factors, iron acquisition, antibiotic resistance mechanisms, and processes related to translation would be notably affected in this adaptive response.

A major result was that growth in WBHV induced significant changes in *A. baumannii* A118 related to translation, with the most enriched pathway for ribosomal protein genes. The upregulation of translation is consistent with previous studies [6,30,31] and reflects how this opportunistic pathogen reacts to the stressful environment of blood. One way that translation upregulation may function is to manage the iron-limiting environment of blood. For example, Eijkelkamp et al. [30] showed that under iron-limiting conditions, 176 genes related to translation were upregulated in *A. baumannii* ATCC 17978. Similarly, Martinez et al. [6] demonstrated that exposing *A. baumannii* to cerebrospinal fluid (CSF) triggered the upregulation of ribosomal protein-associated genes, accounting for 47 out of 55, as well as other pivotal translation genes. Avican et al. [31] discovered “universal stress responders” (USRs), genes with altered expression across various infection-relevant stress conditions, where non-coding RNAs also play a significant role. They identified these responders with high likelihood of gene regulation under specific stress conditions and identified 168 USRs that are pivotal in fundamental biological processes, including ribosome biogenesis.

Notably, growth in human blood impacted some of *A. baumannii* A118’s iron-related pathways, with many genes involved in siderophore biosynthesis and transport upregulated in response to WBHV. It is thought that the regulation of iron levels in blood serves a dual function: not only does it help to counteract the potential toxicity associated with imbalances in iron levels, but it also acts as an innate immune defense mechanism against invading pathogens [7].

Three siderophores have been identified in different strains of *A. baumannii*, namely baumannoferrin(s) (the *bfn* gene cluster), acinetobactin (the *bas/bau* gene cluster and *entA* gene outside this gene cluster), and fimsbactin(s) (the *fbs* gene cluster) [32]. However, acinetobactin is commonly acknowledged as the primary siderophore synthesized by *A. baumannii* due to its pivotal role in *Acinetobacter* pathogenicity [33]. Given previous data showing *A. baumannii* A118 encodes and secretes acinetobactin and baumannoferrin [34], our finding of many upregulated genes for acinetobactin and baumannoferrin biosynthesis and transport in WBHV compared to LB suggest these are part of a key adaptive response of *A. baumannii* through sequestering iron from blood.

Conversely, we found that none of the *feo* genes (encoding the Feo ferrous iron uptake system) were differentially expressed in *A. baumannii* A118 grown in WHBV. The *feo* operon actively transports Fe^2+^ through the cytoplasmic membrane [35,36]. Our results align with the forms of iron available in blood. In the presence of oxygen, iron primarily exists in the oxidized ferric form (Fe^3+^), forming insoluble oxy-hydroxy polymers. Conversely, in anaerobic or reducing environments, iron is mostly in the more soluble ferrous form (Fe^2^) [37]. Absence of *feo* genes among our DEGs can be explained by the scarcity of Fe^2+^ in blood. Additionally, a recent study revealed that while the Feo system enhances growth in heat-inactivated human serum and aids in resisting human serum, its role in virulence is non-essential [38].

Consistent with the critical acquisition of Fe^3+^ from blood, we observed upregulation in two essential genes (*tonB3* and *exbD3*) from the TonB energy transducing machinery when *A. baumannii* A118 was grown in WBHV. The acquisition of Fe^3+^ by bacterial systems, whether through siderophores or heme uptake, necessitates the TonB energy transducing machinery, which comprises the TonB-ExbB-ExbD protein complex [38]. Only *tonB3* has been deemed essential for *A. baumannii* growth in low-iron media and human serum and plays a role in contributing to virulence in animal models of infection [39]. This finding aligns with our results, supporting the essential function of *tonB3* in the low-iron environment of WBHV.

From the Znu (zinc import system) operon, three genes (*znuA*, *B* and *D*) were upregulated when *A. baumannii* A118 was grown in WBHV, supporting the pathogen’s adaptive response to the host-mediated Zn-restricted environment of WBHV [40]. It has been demonstrated that the immune defense against *A. baumannii* in murine lung models, depends on calprotectin – a zinc chelator – secreted by neutrophils [40].

We manually searched for the expression of selected biofilm and quorum sensing related genes based on previous studies [41–47]. All six genes in the *csu* operon, which encode genes for the synthesis of type I pili in *A. baumannii*, were upregulated in WBHV samples. In the initial stages of *A. baumannii* biofilm formation, the *csu* operon is believed to control both pili formation and bacterial attachment to surfaces [48]. To our knowledge, there are no studies assessing the effects of WBHV on biofilm formation in *A. baumannii*, although other studies have assessed the effects of human serum albumin [49], human pleural fluid [5]. However, all of these have assessed relatively later stages of growth (7 or 8 HPI), in contrast to the 4 HPI time point in the current study, and have reported downregulation of several biofilm associated genes. Exploring the transcriptional changes of these genes in whole blood during later stages of bacterial growth presents an intriguing avenue for investigation.

In our study, we detected downregulation of *bap* (encoding the biofilm-associated protein Bap which helps in adherence to host cells) in WBHV samples, which is consistent with this protein’s involvement in maintaining the mature biofilm structure during later stages of biofilm formation [50]. Murray et al. [8] also detected strong downregulation of *bap* in *A. baumannii* ATCC 17978 grown in mice blood which agrees with our result. Similarly, we observed downregulation of *ata*, encoding the Acinetobacter trimeric autotransporter adhesin (Ata), in WBHV samples. This result is consistent with a previous study showing the production of Ata in *A. baumannii* ATCC 17978 across different growth phases in LB, with the highest levels found during the very early exponential phase, followed by a gradual decline throughout the logarithmic and stationary phases [43].

Our SDS-PAGE and LC-MS/MS results showed a potential impact the stressful environment of whole blood on the regulation of OMPs. OMP extraction was conducted at 8 HPI. By this time, cells have had sufficient opportunity to synthesize, fold, and localize OMPs under the tested conditions and more importantly have gone through post-translational regulation. This ensures a comprehensive view of protein expression, capturing stress-induced or condition-specific proteins that may not be abundantly expressed during exponential growth. We observed downregulation of OMPs in response to growth of *A. baumannii* A118 in WBHV, including Omp38, also known as outer membrane protein A (OmpA), Omp 33–36 and CarO. In *E. coli*, OmpA among other several OMPs has been demonstrated to be downregulated in a σ^E^-dependent manner [51]. σ^E^ is a member of the Group IV σ family, which, similar to many other members of this family, mediates respond to stress in the cell envelope [52]. CarO has also been shown to be downregulated in response to various environmental stresses, including oxidative and osmotic stress [53]. The present study is the first to evaluate the expression of *A. baumannii*’s OMPs in response to the stressful environment of blood. Transcriptomic analysis did not show differences in the gene expression for these genes; thus, further research will be needed to fully understand the impact of this stressful environment and the regulation of OMPs.

Connected with decreased expression of OmpA, Omp 33–36, and CarO in WBHV, we also observed decreased susceptibility to imipenem when *A. baumannii* A118 was grown in WBHV compared to LB. The downregulation of the aforementioned OMPs in WBHV likely limits imipenem uptake, requiring antibiotic higher concentrations to achieve >99.9% bacterial killing compared to LB. Specifically since, CarO is known to play a role in transporting carbapenem antibiotics across the cell membrane [54] and it has been shown that *A. baumannii* carbapenem-resistant strains lack CarO or have deficient CarO [55]. A similar pattern is observed with Omp 33–36, where the expression of this OMP is repressed in *A. baumannii* carbapenem-resistant strains [56]. Zhu et al. [57] demonstrated that the levels of Omp33–36 and OmpA in the tested *A. baumannii* carbapenem-resistant isolates were 3-fold and 3.4-fold lower, respectively, compared to the susceptible counterpart. These findings are consistent with our results, confirming that the downregulation of the three identified OMP proteins in WBHV samples likely contributes to carbapenem resistance.

*A. baumannii* A118 grown in WBHV caused over 11% higher *G. mellonella* larval mortality compared to LB-grown bacteria, suggesting enhanced virulence. We grew bacteria in WBHV and LB for 8 hours before injection, allowing time for the expression of key virulence-related proteins in response to blood, as done for OMP extraction. While *G. mellonella* infection model results are not directly translatable to humans, these results highlight the model’s utility and underscore the impact of growth conditions on pathogenicity.

To assess the impact of blood on other virulence factors, we examined twitching motility and found that sheep blood enhanced motility in *A. baumannii* A118 and the Δ*comM* mutant, but not in the Δ*pilA*, Δ*pilT*, or Δ*pilQ* mutants. These genes are essential for type IV pili (TFP)-mediated motility [58–60], and as shown by our results their deletion impaired movement regardless of media, underscoring their critical role. While the Δ*comM* mutant showed reduced motility overall, it exhibited increased movement on blood-containing plates, suggesting *comM* plays a lesser role in twitching motility than key *pil* genes. It is known that *comM* plays a key role in DNA uptake [61]; however, its involvement in twitching motility of *A. baumannii* remains unclear and warrants further investigation.

In conclusion, this study is the first to comprehensively examine *A. baumannii* A118’s transcriptomic response, OMP expression, antibiotic susceptibility, virulence, and motility in WBHV. We identified key adaptations, including upregulation of ribosomal and iron acquisition genes, and downregulation of OMPs like OmpA, Omp33–36, and CarO—potentially contributing to increased imipenem resistance. Growth in WBHV also enhanced virulence in the *G. mellonella* model. These findings highlight critical mechanisms underlying *A. baumannii* A118’s adaptability and virulence in the bloodstream, with implications for managing hospital-acquired infections.

## Supporting information

S1 Fig MA plots displaying log2 fold changes of each gene over the mean of normalized counts for all the samples. Each dot represents a gene in *A. baumannii* A118 genome. Genes with adjusted p-value less than 0.05, are represented by blue dots. Dots which fall out of the window are plotted as open triangles pointing either up or down.
(TIF)

S2 Fig Principal component analysis (PCA) score plot after collapsing technical replicates. showing that the transcriptome of *A. baumannii* A118 grown in LB compared to WBHV. Each WBHV biological sample had at least two technical replicates.
(TIF)

S3 Fig Upregulated genes of *A. baumannii* A118 related to ribosome biogenesis when grown in WBHV compared to LB. 51 Out of 55 ribosomal genes, were upregulated in response to WBHV.
(TIF)

S4 Fig Upregulated genes of *A. baumannii* A118 related to Zn acquisition when grown in WBHV compared to LB. Out of 5 genes present on the *znu* operon, 3 genes were upregulated in response to WBHV.
(TIF)

S5 Fig Differentially expressed genes of *A. baumannii* A118 related to biofilm and quorum sensing/quenching when grown in WBHV compared to LB. Out of 25 selected genes, 12 genes were differentially expressed response to WBHV.
(TIF)

S6 Fig Twitching motility patterns of *A. baumannii* A118, ΔpilA, ΔpilT, ΔpilQ and ΔcomM. Twitching motility patterns were observed at the agarose/plastic interface, comparing the response to TSA plates containing 5% sheep blood (plates on the left) with the control TSA plate lacking blood (plates on the right). Each plate was inoculated by stabbing through the agarose to the surface of a plastic petri dish followed by incubation at 37°C for 24 h. Each experiment was conducted in triplicates.
(TIF)

S1 Table DEGs from RNA-seq analysis. (XLSX)
