# Supplementary figures and images for: Human whole blood influences the expression of Acinetobacter baumannii genes related to translation and siderophore production
Source: PLoS One. 2025 Jul 24;20(7):e0326330. doi: 10.1371/journal.pone.0326330 (PMC12289009; doi:10.1371/journal.pone.0326330)

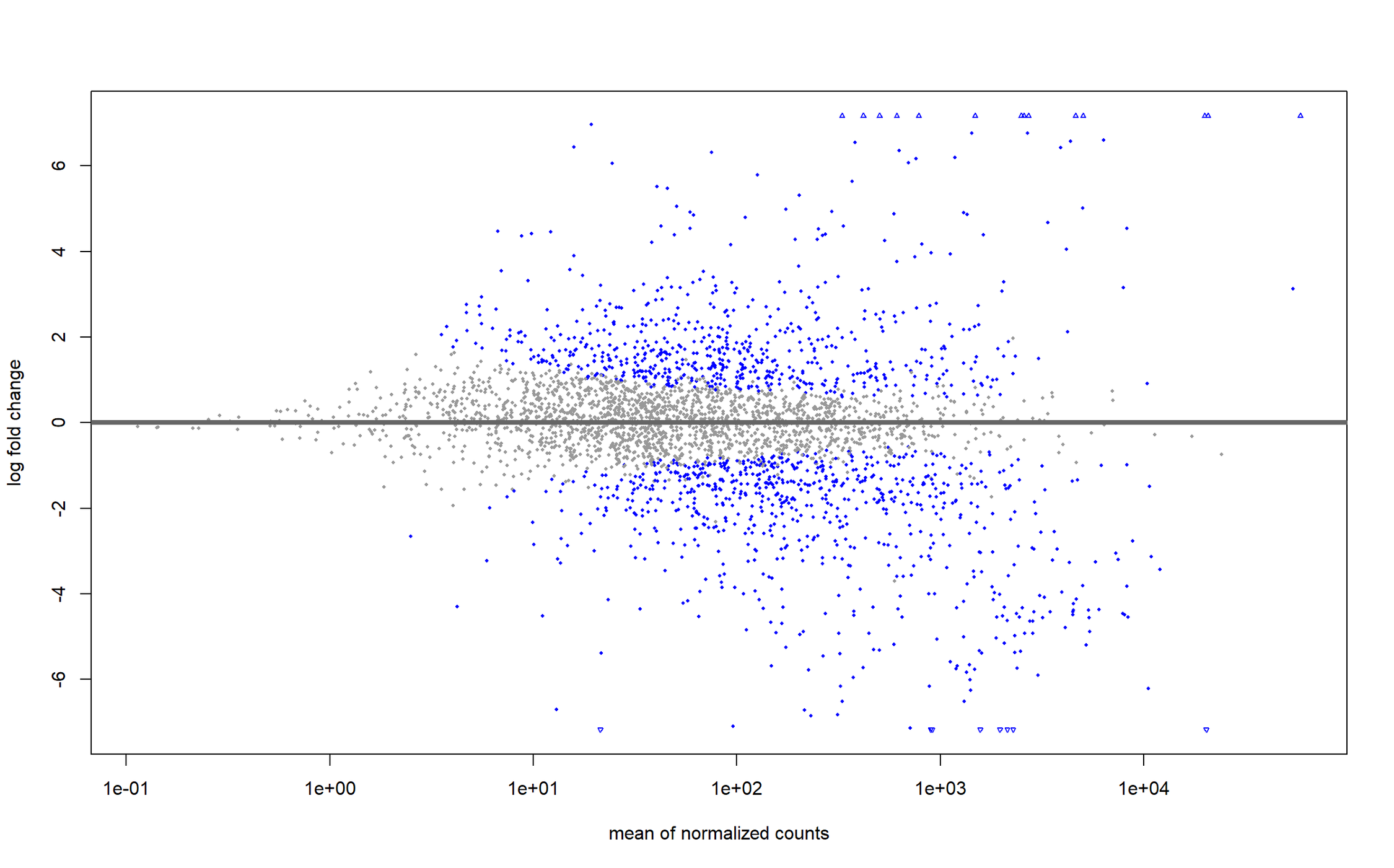

Supplement: S1 Fig — Each dot represents a gene in A. baumannii A118 genome. Genes with adjusted p-value less than 0.05, are represented by blue dots. Dots which fall out of the window are plotted as open triangles pointing either up or down. (TIF) [file pone.0326330.s001.tif]

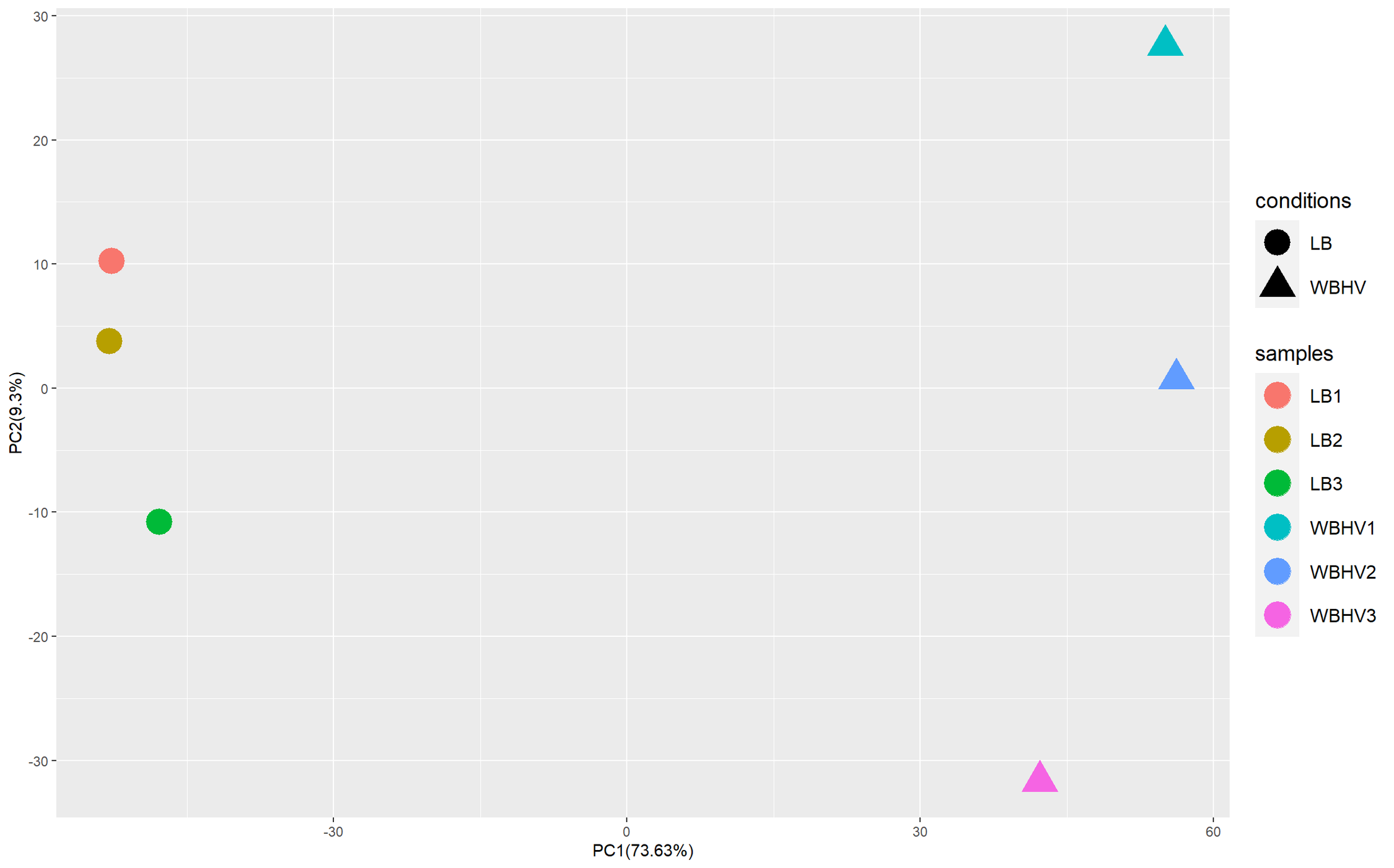

Supplement: S2 Fig — Each WBHV biological sample had at least two technical replicates. (TIF) [file pone.0326330.s002.tif]

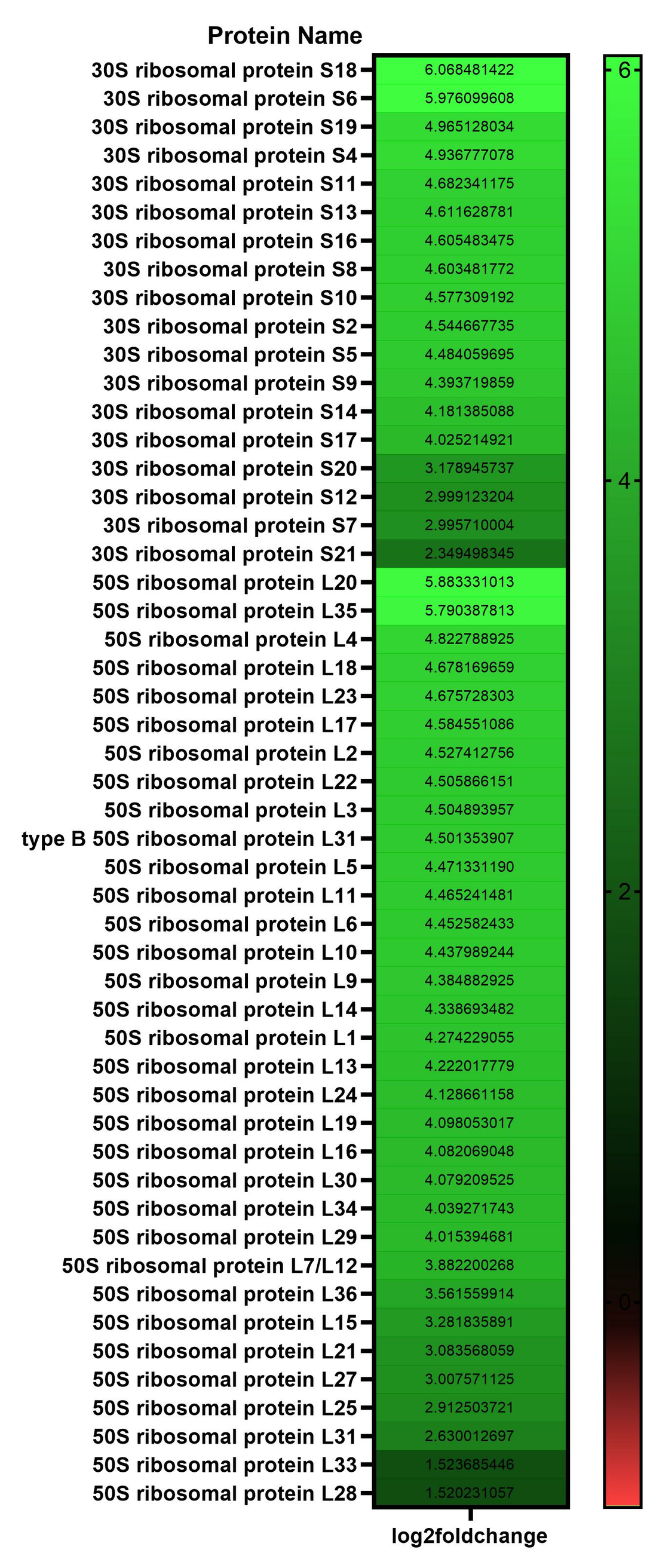

Supplement: S3 Fig — 51 Out of 55 ribosomal genes, were upregulated in response to WBHV. (TIF) [file pone.0326330.s003.tif]

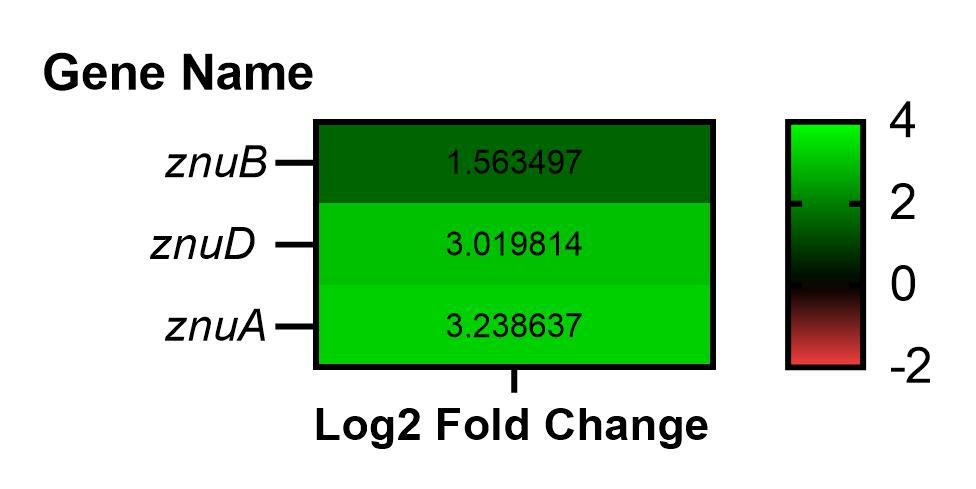

Supplement: S4 Fig — Out of 5 genes present on the znu operon, 3 genes were upregulated in response to WBHV. (TIF) [file pone.0326330.s004.tif]

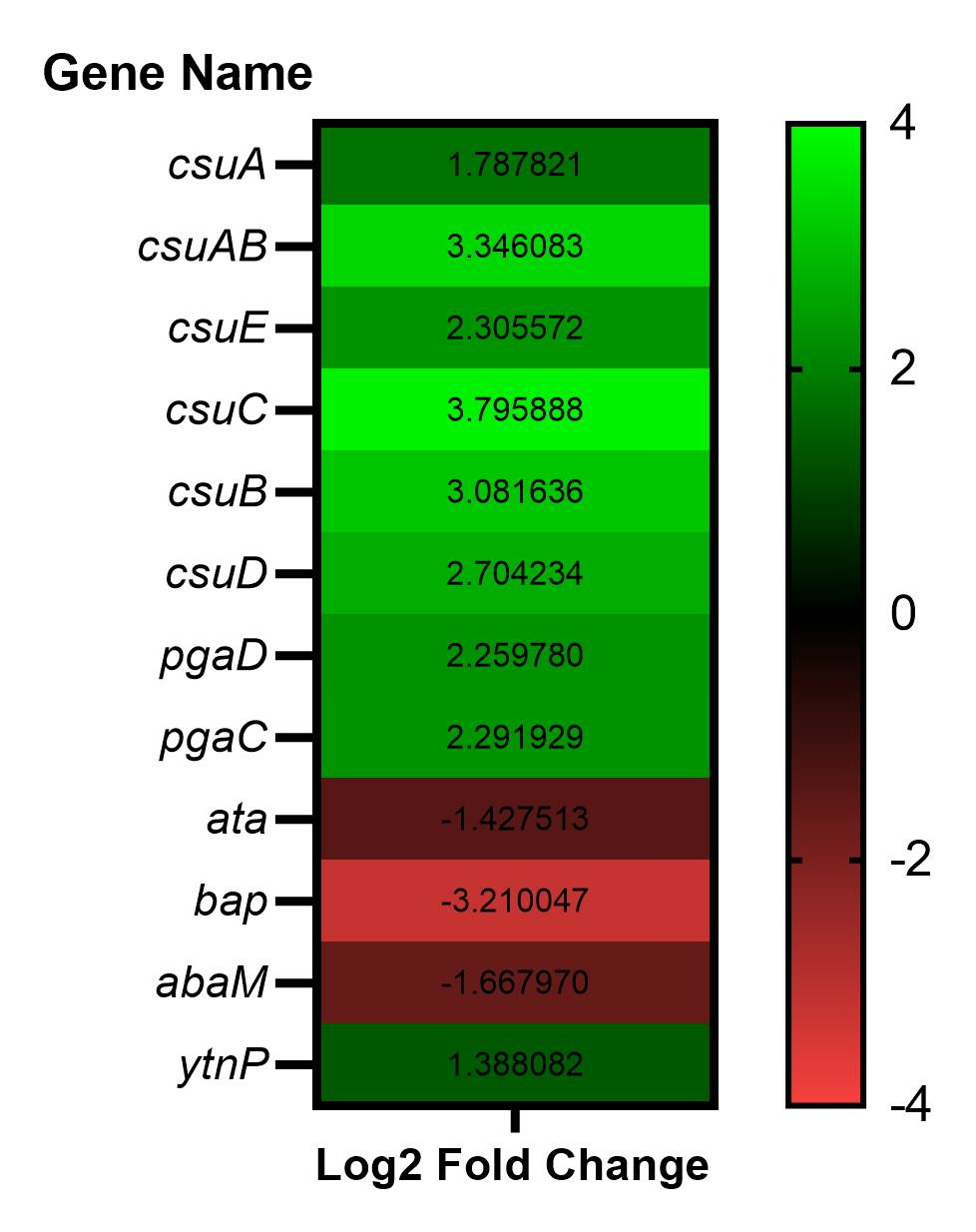

Supplement: S5 Fig — Out of 25 selected genes, 12 genes were differentially expressed response to WBHV. (TIF) [file pone.0326330.s005.tif]

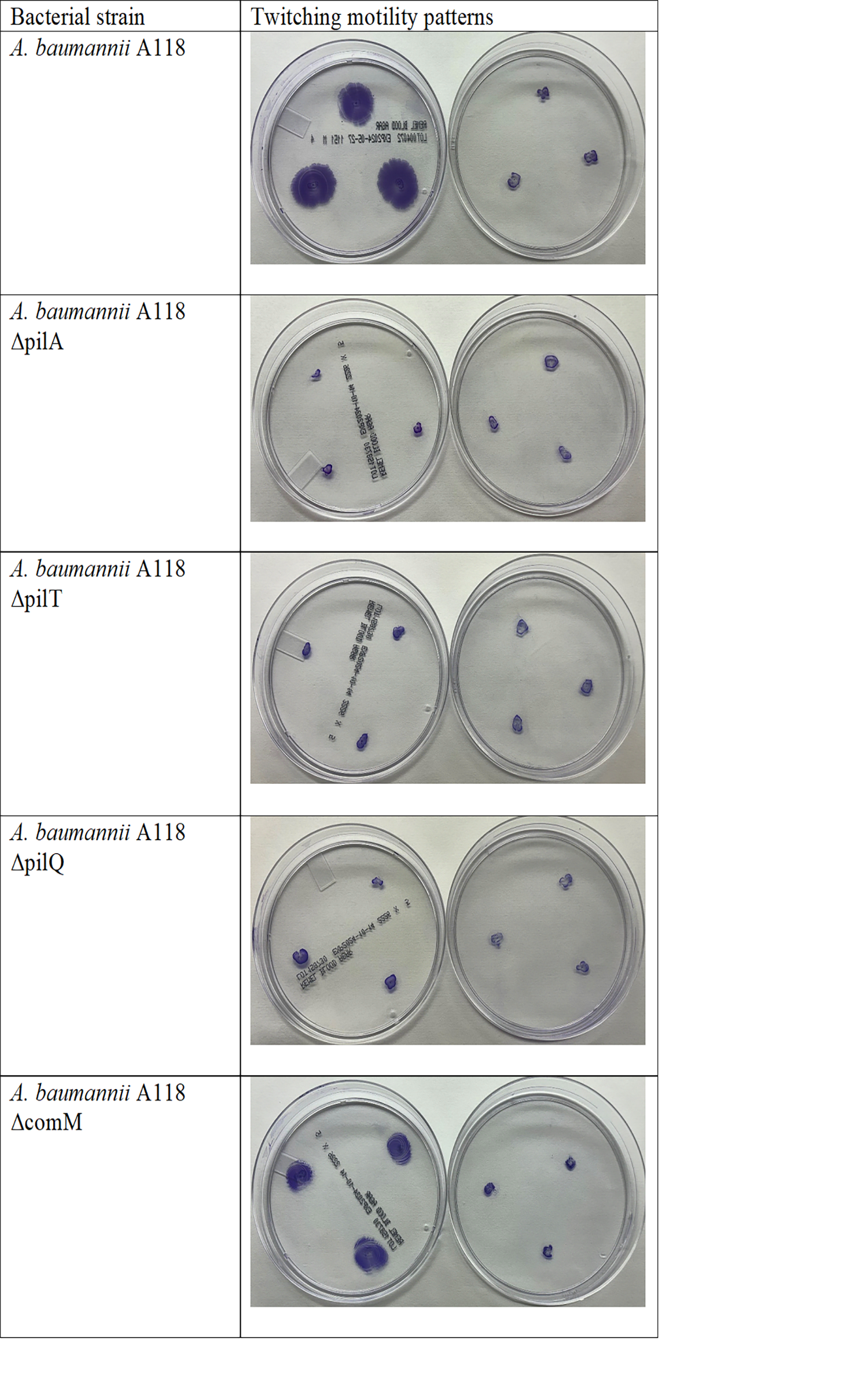

Supplement: S6 Fig — Twitching motility patterns were observed at the agarose/plastic interface, comparing the response to TSA plates containing 5% sheep blood (plates on the left) with the control TSA plate lacking blood (plates on the right). Each plate was inoculated by stabbing through the agarose to the surface of a plastic petri dish followed by incubation at 37°C for 24 h. Each experiment was conducted in triplicates. (TIF) [file pone.0326330.s006.tif]
